# Accuracy and Efficiency of the Surgical-Guide-Assisted Fiber Post Removal Technique for Anterior Teeth: An Ex Vivo Study

**DOI:** 10.3390/dj12100333

**Published:** 2024-10-18

**Authors:** Ryota Ito, Satoshi Watanabe, Kazuhisa Satake, Ryuma Saito, Takashi Okiji

**Affiliations:** Department of Pulp Biology and Endodontics, Division of Oral Health Sciences, Graduate School of Medical and Dental Sciences, Tokyo Medical and Dental University (TMDU), 1-5-45 Yushima, Bunkyo-ku, Tokyo 113-8549, Japan; itohendo@tmd.ac.jp (R.I.); takendo@tmd.ac.jp (K.S.); iyud3389@tmd.ac.jp (R.S.); t.okiji.endo@tmd.ac.jp (T.O.)

**Keywords:** anterior teeth, fiber post, guided endodontics, intra-radicular reinforcement, operator experience, post removal, surgical guide

## Abstract

Background/Objectives: This study compared the accuracy and efficiency of different surgical-guide (SG)-assisted and freehand drilling techniques for removing fiber posts from maxillary anterior teeth performed by differently experienced operators. Methods: A fiber post was bonded to the root canal of 54 extracted maxillary anterior teeth. After mounting the teeth in the jaw models, SGs were designed by integrating cone-beam computed tomography (CBCT) and intraoral scanner data. Each SG included a custom sleeve. An experienced or inexperienced operator drilled the post using three different techniques: (i) SG-assisted incremental drilling at 2–3 mm (SG1), (ii) SG-assisted one-time drilling to a predetermined depth (SG2), and (iii) freehand incremental drilling without SG (FH; n = 9 in each group). Deviations in coronal, sagittal, and horizontal planes and the angle of deviation were measured. Results: The SG1 and SG2 groups showed significantly smaller sagittal and horizontal deviations than the FH group, regardless of the operator’s experience. The SG2 group had a significantly shorter working time than the SG1 and FH groups. In the FH group, the experienced operator required a significantly shorter working time than the inexperienced operator. Conclusions: SG-assisted drilling techniques enhanced the accuracy and efficiency of removing fiber posts from the anterior teeth, irrespective of the operator’s experience.

## 1. Introduction

In recent years, composite resin cores with fiber posts have been widely used to restore root-canal-treated teeth owing to advancements in dental adhesive technologies [1]. Fiber posts are crucial for retaining the coronal restoration through providing internal support to the restoration material and are thought to reduce the risk of root fractures because their flexural modulus is similar to that of dentin, allowing for uniform stress distribution across the root [2]. When a large amount of the coronal tooth structure remains in the root-filled anterior teeth, particularly those with a wide root canal space, intra-radicular reinforcement using fiber posts may be performed to improve the fracture strength of the cervical and root dentin [3,4].

When nonsurgical root canal retreatment is required in teeth restored using fiber posts, they should be removed to regain access to the root canal system [5]. Removing the fiber post typically involves the use of a bur or an ultrasonic tip [6]; however, such a procedure can be challenging because of the color of the fiber post, which closely resembles that of dentin, even under a dental operating microscope [7]. Moreover, the three-dimensional direction of fiber posts in root canals may not be predictably determined using conventional periapical X-ray images [8]. Actual clinical recognition of the axial cutting direction may also be inaccurate despite the aid of cone-beam computed tomography (CBCT) [9]. Still, despite the use of dental operating microscopes, fiber post removal may cause excessive dentin loss and deviation from the original root canal [10], which may reduce the efficacy of root canal cleaning [11,12] and the success rate of endodontic retreatment [13]. Operator experience is considered crucial for reducing the risk of iatrogenic damage, such as aberrant drilling and root perforation, during fiber post removal [14]; thus, alternative techniques are required that enable inexperienced operators to remove fiber posts safely and efficiently.

Recently, the integration of digital technology into dentistry has gained popularity, with global efforts focused on enhancing the safety of dental treatment. This involves the use of digital tools that combine computer-aided design and computer-aided manufacturing (CAD/CAM) systems with imaging data from CBCT [15,16,17,18,19,20,21,22]. For instance, surgical guides (SGs) ensure the accurate and safe placement of implants [15], and dynamic navigation systems offer real-time, three-dimensional feedback on the handpiece position to assist in drilling [16]. In endodontics, guide-assisted techniques incorporating digital technologies, such as CBCT, intraoral scanners, and CAD/CAM, are gaining prominence [17,18,19,20,21,22,23,24,25,26,27]. These techniques are broadly categorized into static guides, such as SG [17,18,23,24,25,26], and dynamic guides, such as the dynamic navigation system [19,20,21,22]. Their applications include access cavity preparation in teeth with pulp canal obliteration [17,18,19,20,21,23] and osteotomy and root resection for endodontic microsurgery [22], demonstrating their vital role in enhancing accuracy and improving outcomes in complex dental procedures.

However, there are few reports on the accuracy and efficiency of SG-assisted drilling techniques for fiber post removal [24,25,26,27], and their clinical applications have not yet been established. In particular, the procedure for using SGs in fiber post removal has not been standardized and varies across studies. For example, the root canal has been assessed for accessibility to the gutta-percha and cleaned every 5 mm [24] or after a specified number of times (2–3 times) [25,26]. This gap in knowledge highlights the need for further investigation into the effectiveness of fiber post removal techniques and the application of SGs across various methodologies. Additionally, most studies on fiber post removal have focused on the premolars [24,25] and molars [24], with limited research involving anterior teeth having a remaining crown [26,27], where correct recognition of the drilling direction can be more challenging due to the greater inclination angle of the tooth axis in maxillary anterior teeth [28].

Therefore, this study aimed to compare the accuracy and efficiency of different SG-assisted and freehand drilling techniques for the removal of fiber posts fixed for intra-radicular reinforcement of maxillary anterior teeth with a remaining crown, performed by operators with different levels of clinical experience. The deviation of the drilled pathway from the planned pathway and the working time required for drilling were compared. The present experimental condition could offer valuable evidence that SG-assisted drilling techniques improve the accuracy and efficacy of fiber post removal regardless of the operator’s experience. The null hypotheses were that (i) SG-assisted drilling techniques do not enhance the accuracy and efficiency of fiber post removal compared to freehand removal, and (ii) the operator’s experience does not influence the accuracy and efficiency of fiber post removal.

## 2. Materials and Methods

### 2.1. Sample Size

Sample size calculations were performed using the G*Power software, version 3.1.9 (Heinrich-Heine Universität, Düsseldorf, Germany). Following a previous study conducted with an effect size of 0.4 and a power of 0.8 [29], the number of samples required was estimated to be 54.

### 2.2. Tooth Selection

Fifty-four extracted human maxillary anterior teeth (18 central incisors, 18 lateral incisors, and 18 canines) were used in this study (approved by the Institutional Review Board of Tokyo Medical and Dental University, No. D 2022-033). The teeth were extracted for reasons unrelated to this study, and informed consent was obtained from all donors. The teeth were stored at 5 °C with 100% relative humidity. All the studied teeth were free of caries and restorations. They also had a completely formed root and a straight root canal, with less than 20° of root curvature according to Schneider’s classification [30], with root length exceeding 15 mm. Teeth that had been previously treated with a root canal, had cracks or fractures, or showed severe signs of attrition and abrasion in the crown were excluded from the study.

### 2.3. Sample Preparations

A single operator prepared an access cavity with a diamond point (smooth-cut AR2f, GC, Tokyo, Japan) and confirmed the patency using a size 10 K file (Dentsply Sirona, Ballaigues, Switzerland) by visually inspecting the file tip from the apical foramen. The working length was set at 1 mm below the apical foramen. The root canals were instrumented to size 35 and 0.06 taper using nickel-titanium rotary instruments (Voltex Blue, Dentsply Sirona) rotated at 500 rpm, during which the root canals were irrigated with 2.5 mL of 3% sodium hypochlorite solution (Dental Antiformin, Nippon Shika Yakuhin, Shimonoseki, Japan). After instrumentation, the root canals were dried with paper points (Dentsply Sirona) and filled with gutta-percha points (Morita, Tokyo, Japan) and a zinc-oxide non-eugenol root canal sealer (Canals N, GC Showa Yakuhin, Tokyo, Japan) using a matched cone technique. After root canal filling, the teeth were sealed with temporary filling material (Caviton EX, GC, Tokyo, Japan).

After 24 h of storage of the teeth at 37 °C with 100% humidity, standardized post-space preparation was performed to a depth of 8.5 mm from the incisal edge using a Peeso reamer (#3, φ1.10 mm; Mani, Tochigi, Japan), followed by a post-preparation drill (RTP, #2, φ1.17 mm; Dentech, Tokyo, Japan) rotated at 10,000 rpm with a contra-angle electric handpiece (EXPERTmatic LUX E20L, Kavo, Biberach, Germany) without water spray. The cavities were rinsed with 2.5 mL of water and dried using #60 paper points. Post-cementation was performed according to the manufacturer’s instructions. A universal-type bonding agent (Clearfil Universal Bond Quick EQ; Kuraray Noritake Dental, Tokyo, Japan) was applied to the root canal wall with a micro-brush, dried with a mild air blow, and light-cured with a codeless light-curing unit (Pen Cure 2000, Morita, Tokyo, Japan) in the standard mode (1000 mW/cm^2^) for 20 s. A glass fiber post (GC Fiber Post N φ1.0 mm, GC), cut 7.5 mm-long and treated with a silane coupling agent (Clearfil Ceramic Primer, Kuraray Noritake Dental), was placed into the full depth of the post-space filled with a dual-cure composite resin for core build-up (Clearfil DC Core Automix, Kuraray Noritake Dental). Excess resin was removed, and the resin was light-cured for 20 s. The samples were stored at 37 °C with 100% humidity for 7 days.

**Figure 1:**
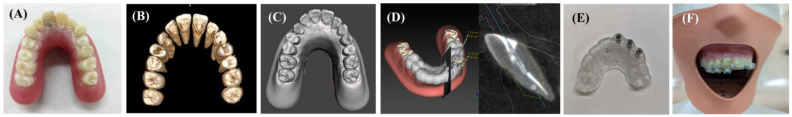
Three-dimensional planning, fabrication, and application of the surgical guide for fiber post removal. (**A**) Dental jaw model. (**B**) Preoperative CBCT image, 3D reconstruction. (**C**) Preoperative optical scan image. (**D**) Implant planning software (DTX Studio, version 3.5.5.1) was used for drilling planning after merging the CBCT and intraoral scanning data. (**E**) Fabricated surgical guide. (**F**) The model was attached to a dental phantom head and fiber post removal was performed.

Next, the teeth were mounted on maxillary jaw models (Wax Form DN3-RM19, JM Ortho Corporation, Tokyo, Japan) according to tooth type, and positioned with an average tooth axis inclination of approximately 60°, corresponding to the jaw model socket [31]. The intact extracted molars were used as references (Figure 1A).

### 2.4. Designing and Fabrication of the SGs

Digital imaging and communication in medicine (DICOM) data of the jaw model were obtained by CBCT imaging (3D Accuitomo F17D, Morita, Tokyo, Japan; Figure 1B) under settings of 90 kV and 8.0 mA, with an imaging range of 80 mm × 80 mm, and a voxel size of 160 µm. Standard tessellation language (STL) data for the jaw model were obtained using a three-dimensional intraoral scanner (E1, 3shape; Copenhagen, Denmark; Figure 1C). The SGs were designed by integrating the DICOM and STL data into implant simulation software (DTX Studio software, Nobel Biocare, Zürich, Switzerland; Figure 1D). Since the software is designed for implants, the thinnest implant model was used to simulate the SG sleeves (outer/inner diameter: φ3.0/1.5 mm and length: 10 mm). The SG was designed with three sleeves to drill the maxillary central incisor, lateral incisor, and canine on one side. However, the corresponding teeth on the opposite side were drilled freehand. The design data were sent to Nobel Biocare for SG fabrication (Figure 1E).

For guide diameter compensation, a custom-made metal sleeve (outer/inner diameter: φ1.50/1.18 mm × height 4.6 mm, general tolerance: ±0.03–0.05; Yasuhisa Koki, Tokyo, Japan), made of stainless steel (SUS304), was inserted into the existing metal sleeve (inner diameter: 1.50 mm) and fixed with a cyanoacrylate adhesive (Aron Alpha 201, Toa Gosei, Tokyo, Japan). This modification resulted in the creation of a custom-made SG specifically designed for fiber post removal, with an inner sleeve diameter of 1.18 mm. The diameter of the bur used in drilling was 1.0 mm, so the play between the bur and sleeve was 0.18 mm.

### 2.5. Fiber Post Removal

The jaw models were randomly divided into 3 groups according to the removal methods, as described below, and each group was subdivided according to the operator’s clinical experience (an experienced operator with 15 years of clinical experience, mainly in endodontic treatment, or an inexperienced operator with 1 year of clinical experience; n = 9 teeth in each subgroup). The jaw model was attached to a dental phantom head (NISSIN Type 1 Plus, Nissin, Kyoto, Japan; Figure 1F). The drilling procedure was carried out using a long-shaft carbide round bur (Munce Discovery Burs #2, φ1.0 mm, 34 mm in length; CJM Engineering Inc., Ojai, CA, USA) rotated at 10,000 rpm with a contra-angle electric handpiece (EXPERTmatic LUX E20L, Kavo, Biberach, Germany) without water spray. The working time required for drilling was measured using a stopwatch.

SG-assisted post removal was performed by advancing a rotating bur inserted through the sleeve in the apical direction under light pressure. In the SG1 group, the bur was incrementally advanced in 2–3 mm steps. Between each step, the canal was irrigated with tap water and inspected using a dental operating microscope (OPMI Pico, Carl Zeiss, Oberkochen, Germany) at 8.2× magnification. In contrast, the SG2 group involved a one-time bulk drilling directly to the predetermined depth at which the end of the fiber post was located. In the freehand (FH) group, the SG was not used for the drilling procedure, the bur was incrementally advanced, and the canal was irrigated and inspected, as in the SG1 group. In the FH and SG1 groups, drilling was stopped upon visual confirmation of the gutta-percha, whereas in the SG2 group, drilling was stopped once the predetermined depth was reached. The gutta-percha was visible in all samples in the SG2 group. The drilling procedure was performed from the central incisor to the canine, alternating between the SG and FH. To ensure uniform proficiency, each operator preliminary practiced the drilling on three teeth across all groups before commencing the study.

### 2.6. Data Analysis

**Figure 2:**
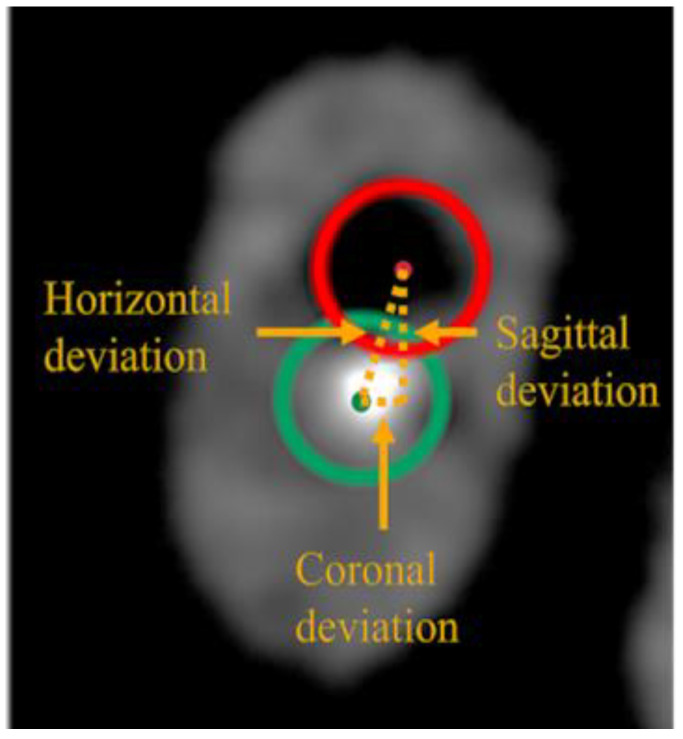
Measurement of the deviations. Deviations between the center of the planned (green) pathway and the drilled (red) pathway in the coronal, sagittal, and horizontal planes were measured on a DICOM viewer (RadiAnt DICOM Viewer).

After drilling, each specimen was subjected to a post-operative CBCT scan with the same parameters as the preoperative scan. The pre- and post-operative CBCT images were superimposed, and measurements were performed on a DICOM viewer (RadiAnt DICOM Viewer, Medixant, Poznań, Poland). Deviations between the centers of the planned and drilled pathways in the coronal, sagittal, and horizontal planes were measured at 1 mm short of the end of the planned pathway using the superimposed horizontal image of this level (Figure 2). The angle of deviation was determined by measuring the angle between the central axis of the planned drilling trajectory and the post-drilling trajectory. Following the superimposition of the trajectories on the coronal and sagittal images, the maximum value was adopted.

### 2.7. Statistical Analysis

The collected data were analyzed using statistical analysis software (SPSS version 28.0, SPSS Inc., Chicago, IL, USA). As the Shapiro–Wilk test did not verify data normality, the Kruskal–Wallis test and Mann–Whitney U test with Bonferroni correction for multiple comparisons were used to analyze the amount of deviation, deviation angle, and time required for drilling in each group. The significance level was set at α = 0.05.

## 3. Results

**Figure 3:**
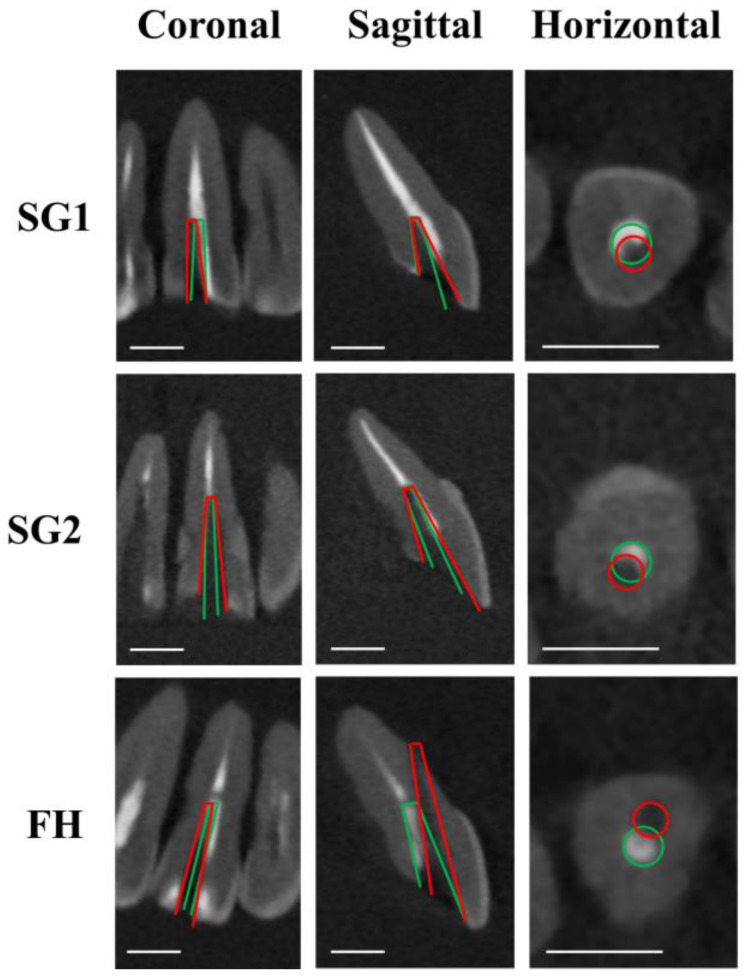
Representative CBCT images after fiber post removal on central incisors. Green lines show planned pathways, and red lines show actual drilling pathways. Scale bar = 5 mm.

**Figure 4:**
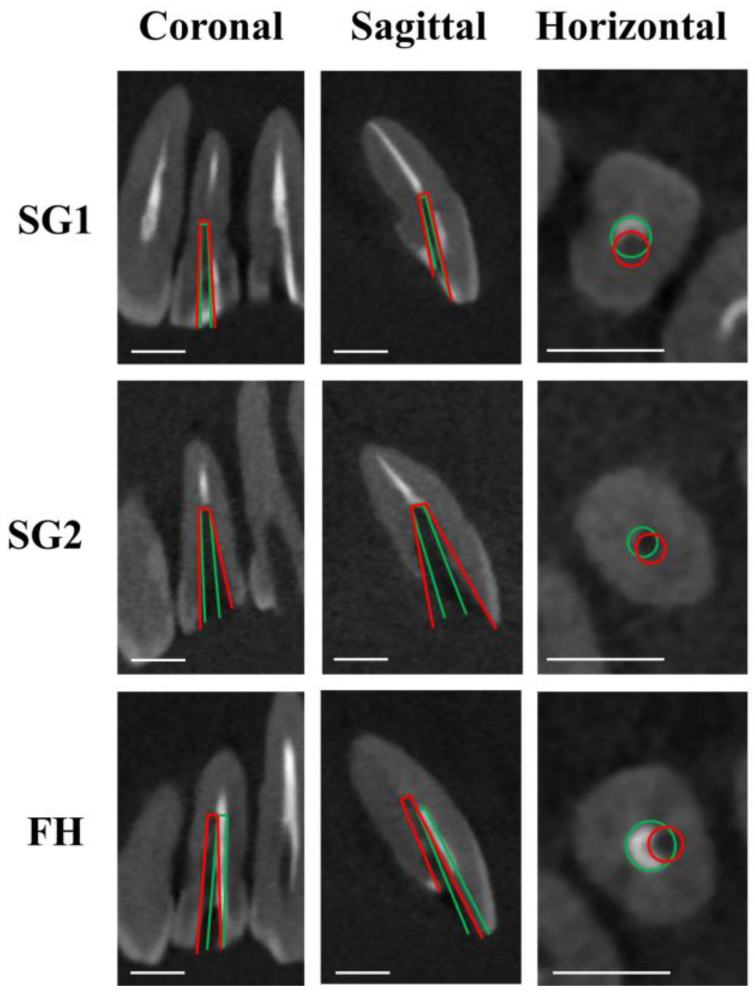
Representative CBCT images after fiber post removal on lateral incisors. Green lines show planned pathways, and red lines show actual drilling pathways. Scale bar = 5 mm.

**Figure 5:**
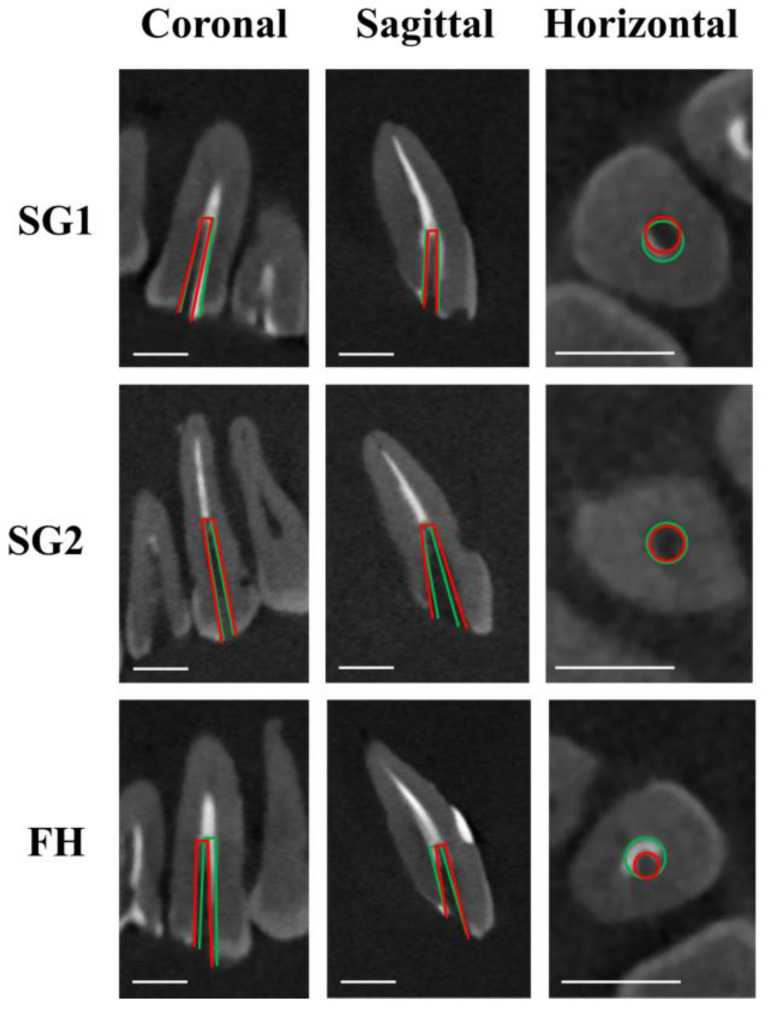
Representative CBCT images after fiber post removal on canines. Green lines show planned pathways, and red lines show actual drilling pathways. Scale bar = 5 mm.

Figure 3, Figure 4 and Figure 5 illustrate the representative CBCT images before and after fiber post drilling in each group. Table 1 presents the amount of deviation in the coronal, sagittal, and horizontal planes, the angle of deviation, and the working time required.

Both SG-assisted groups (SG1 and SG2) showed significantly smaller deviations and angles of deviation in the sagittal and horizontal planes than the FH group, regardless of the operator’s experience (*p* < 0.05). For the experienced operator, there was no significant difference in the degree of deviation in the coronal plane between the SG2 and FH groups (*p* > 0.05). There was also no significant difference between the SG1 and SG2 groups in any plane (*p* > 0.05).

In the SG1 and SG2 groups, all specimens were drilled to the expected position, where the gutta-percha was exposed without excess drilling in the apical direction. Root wall perforation occurred in one tooth in the FH group, which was performed by an inexperienced operator. In contrast, the experienced operator did not cause perforation in any of the groups.

The working time required for drilling was significantly shorter for the experienced operator than for the inexperienced operator (*p* < 0.05) in the FH group. In contrast, there was no significant difference between the operators in the SG group (*p* > 0.05). The SG2 group was associated with a significantly shorter working time than the SG1 and FH groups (*p* < 0.05).

## 4. Discussion

In recent years, fiber posts have been increasingly used as the material for root canal posts instead of prefabricated or cast metal posts [1]. Fiber posts have a flexural modulus similar to that of dentin, which allows them to deflect with the root when a force is applied, thereby reducing the stress concentration in the root [2]. However, the removal of fiber posts may pose challenges for nonsurgical endodontic retreatment. As indicated in some studies [24,25,26,27,32], drilling with the aid of SGs may be promising for the safe removal of intracanal fiber posts. However, a standardized drilling technique using SGs has not yet been established, and various techniques have been employed in different studies [24,25,26,32]. Thus, this study compared the accuracy and efficiency of fiber post removal using two SG-assisted techniques, incremental and one-time, and the conventional FH technique. This study also compared the performances of experienced and inexperienced operators using these techniques to investigate how operators with different levels of experience benefit from SG-assisted fiber post removal.

The first null hypothesis of this study was rejected because the SG group showed a significantly smaller angle of deviation than the FH group, and the SG2 group was associated with a shorter working time than the other groups. The second hypothesis was partly rejected because a significant difference was detected only for working time in the FH group, in which the experienced operator took a shorter time than the inexperienced operator.

To the best of our knowledge, this is the first study to evaluate the accuracy and efficiency of two SG-assisted fiber post removal techniques involving anterior teeth having a remaining crown to which a fiber post was placed for intra-radicular reinforcement. This condition can be regarded as simulating challenging cases because the tooth axis inclination angle of the anterior teeth (approximately 60–70° from the occlusal plane) was larger than that of the molars (approximately 80°) [31], which led to a higher likelihood of drilling deviation to the labial side [28]. In addition, post lengths generally tend to be longer in anterior teeth, making removal more difficult than in molars, as post length is often determined based on the crown length, which is longer in anterior teeth [33].

In this study, simulation software was used for dental implant SG fabrication because there is currently no dedicated software for SGs, specifically for endodontic applications. The drilling trajectory was designed for the smallest-diameter implants. The inner diameter of the metal sleeve for the smallest-diameter pilot drill was 1.5 mm, whereas the long-shaft carbide bur used in this study had a diameter of 1.0 mm, which resulted in a gap of approximately 0.5 mm. When the bur was inserted into the sleeve at its deepest point without any modification, a significant gap was observed at the tip of the bur, preventing accurate drilling. For this reason, a custom-made metal sleeve (outer/inner diameter/length: φ1.50 mm/φ1.18 mm/4.6 mm; general tolerance: ±0.03–0.05) made of stainless steel (SUS304) was attached to the inner surface of the existing sleeve to minimize the gap between the bur and the sleeve as much as possible. In previous studies [24,25], the diameter of the bur and the inner diameter of the sleeve were designed to be the same (0.75 mm). However, if the inner diameter of the metal sleeve and the diameter of the bur were identical, there would be no gap, potentially preventing the bur from rotating and hindering the discharge of drilling debris, particularly in the bulk drilling group (SG2). To address this issue, a minimum gap of 0.18 mm was used to maintain the rotation, facilitate drilling debris discharge, and minimize blurring. As the actual amount of deviation was equivalent to that reported in a previous study [34], the 0.18 mm gap may not have affected the accuracy of the results. A previous study [26] investigated the accuracy of using a single sleeve or multiple sleeves in a single surgical guide and found no difference in accuracy. Therefore, multiple sleeves were placed in a single surgical guide in this study as well.

Regarding the experienced operator, the SG group showed significantly less deviation than the FH group in the sagittal section. In contrast, there was an unexpected finding of no significant difference between the SG2 and FH groups in the coronal section. This discrepancy may have arisen because when drilling with the FH, the mesiodistal deviation is relatively easy to recognize and control; however, controlling the drilling operation becomes particularly challenging in the buccolingual direction, especially when the tooth axis inclination is large [21,28]. In contrast, previous studies using premolars and molars showed no significant differences in coronal sections [24,25]. This can be explained as follows: In molars, the crown and root axes are relatively coincident, and the tooth axis inclination angle is small [28], which may facilitate the recognition of buccolingual displacement. Thus, the tendency for labial displacement in the sagittal section is presumed to be specific to the anterior teeth.

Regarding the inexperienced operator, the use of SGs resulted in a significantly smaller amount of deviation in all cross-sections compared with FH drilling. Both SG groups showed significantly less deviation in the sagittal and horizontal sections than the FH group. These findings are similar to those reported in a previous study [25] and indicate that the use of SGs helps prevent accidental injuries, such as excessive drilling of the tooth structure and root perforation during fiber post removal. The amount of deviation observed in this study was also comparable to those reported in previous studies, showing a deviation of 0.24 to 0.40 mm after fiber post removal using SG [24] and 0.17 to 0.47 mm after access cavity preparation in teeth with a constricted canal [34]. Regarding the deviation angle, both SG-assisted groups demonstrated significantly smaller values than the FH group, which is in line with previous reports [24,34] and indicates that the bur accurately followed the pre-simulated trajectory through the SG. The results also showed that the same level of drilling accuracy was achieved regardless of the operator’s years of experience. Thus, it is reasonable to suppose that SG-assisted fiber post removal may enable any practitioner to achieve high-precision drilling while minimizing the risk of iatrogenic events, such as overzealous drilling and root perforation.

The working time required for the groups was significantly shorter in the SG2 group than in the SG1 and FH groups. This can be explained by the fact that SG-assisted one-time drilling improved efficiency by eliminating repeated interruptions accompanied by irrigation and microscopic inspection during the drilling procedure. It was also noted that SG1 did not always lead to a working time reduction, particularly in the experienced operator, who was most probably skillful enough to efficiently remove fiber posts with FH drilling. In the FH group, the inexperienced operator required a significantly longer working time than the experienced operator, whereas in the SG group, there were no significant differences in working time between experienced and inexperienced operators. Moreover, root perforation only occurred after FH drilling by the inexperienced operator. These findings clearly indicate that SG is particularly beneficial for inexperienced dentists who are not accustomed to fiber post removal by FH. Similar benefits have been reported with the use of SGs for implants [35]. SGs are also anticipated to be effective tools for accurate fiber post removal in settings where dental microscopes are unavailable.

In addition to a static surgical guide, a dynamic navigation system that offers real-time feedback on the drilling position and allows for trajectory corrections during drilling has been utilized for guided endodontic procedures [19,20,21,22,29,36]. In a study on the removal of fiber posts, both the surgical guide and dynamic navigation systems proved to be equally effective and accurate, surpassing the results of freehand techniques [29]. However, the effectiveness of dynamic navigation systems is notably influenced by the operator’s experience [29]. In contrast, although the surgical guide does not permit trajectory corrections during drilling, it clearly enables accurate drilling without relying on operator experience by following the direction determined by the predesigned sleeve [37].

One limitation of this study is that it did not consider the accumulation of drilling debris, particularly in the SG2 group. However, it seems reasonable to suppose that increasing the inner diameter of the sleeve to facilitate irrigation and debris removal may make the guided bur unstable, thereby significantly reducing the accuracy. Additionally, this study used different tooth types on both sides, which could influence the results due to variations in the angle of inclination and left and right differences in factors, such as the sight direction and location of the working area during drilling. Moreover, concerns exist regarding the effect of the heat generated during drilling on the periodontal ligament and surrounding alveolar bone. Future research should focus on developing and evaluating protocols for SG-assisted drilling that employ appropriate SG size and cooling methods while accounting for tooth types, axis inclinations, and post lengths.

## 5. Conclusions

Both SG-assisted fiber post removal techniques improved the accuracy, with less deviation than FH. Moreover, the one-time drilling technique with SG significantly reduced the working time, independent of the operator’s experience. These results indicate that the SG-assisted drilling techniques not only improved the accuracy but also the efficiency of removing fiber posts from the anterior teeth, offering significant benefits to both inexperienced and experienced operators.

## Figures and Tables

**Table 1:** Deviations in each cross-section, angle of deviation, and working time.

	Operator	Group		
		SG1	SG2	FH
Coronal (mm)	Experienced	0.23 (0.21–0.45) ^A^	0.38 (0.25–0.39) ^AB^	0.59 (0.42–0.71) ^B^
	Inexperienced	0.33 (0.28–0.42) ^A^	0.33 (0.23–0.48) ^A^	0.55 (0.45–0.83) ^B^
Sagittal (mm)	Experienced	0.23 (0.20–0.47) ^A^	0.44 (0.31–0.53) ^A^	0.92 (0.66–1.25) ^B^
	Inexperienced	0.32 (0.28–0.56) ^A^	0.41 (0.40–0.48) ^A^	1.41 (0.48–1.66) ^B^
Horizontal (mm)	Experienced	0.43(0.32–0.66) ^A^	0.60 (0.57–0.74) ^A^	0.92 (0.81–1.32) ^B^
	Inexperienced	0.54 (0.49–0.67) ^A^	0.53 (0.51–0.72) ^A^	1.32 (0.60–1.49) ^B^
Angle (degree)	Experienced	2.48 (2.33–3.66) ^A^	3.34 (2.35–3.80) ^A^	5.87 (4.92–8.46) ^B^
	Inexperienced	2.56 (2.31–3.60) ^A^	2.75 (2.33–3.10) ^A^	8.22 (6.96–9.58) ^B^
Working Time (s)	Experienced	139.0 (87.0–282.0) ^A^	29.0 (27.0–32.0) ^B^	91.0 (69.0–122.0) ^A#^
	Inexperienced	141.0 (111.0–201.0) ^A^	29.0 (26.0–35.0) ^B^	278.0 (164.0–421.0) ^A#^

Data represent the median and interquartile range (n = 9). Different superscript letters in the same row indicate statistically significant differences (*p* < 0.05). ^#^ Significant differences by operator experience in the same drilling method (*p* < 0.05).

## Data Availability

The data presented in this study are available upon request from the corresponding author.
